# Glycaemia as a sign of the viability of the foetuses in the last days of gestation in dairy goats with pregnancy toxaemia

**DOI:** 10.1186/2046-0481-65-1

**Published:** 2012-01-23

**Authors:** Miguel S Lima, Rita A Pascoal, George T Stilwell

**Affiliations:** 1 DC, CIISA, Faculdade de Medicina Veterinária, Universidade Técnica de Lisboa, Polo Universitário da Ajuda, Lisboa, 1300 - 477, Portugal; 2 Barão e Barão, Coutada Velha, Benavente, 2130 - 010, Portugal; 3 AWIN - Animal Wellfare Indicators, Faculdade de Medicina Veterinária, Universidade Técnica de Lisboa, Polo Universitário da Ajuda, Lisboa, 1300 - 477, Portugal

**Keywords:** hyperglycemia, pregnancy toxemia, goats

## Abstract

Pregnancy toxaemia is one of the most common diseases affecting small ruminants in the last month of gestation. Nearly 80% of the foetal growth occurs in the last 6 weeks of gestation. Fat goats and goats carrying twins and triplets are at greater risk. Pregnancy toxaemia is characterized by metabolic acidosis, hypoglycaemia and ketonaemia and a very high mortality rate. In our study five does with pregnancy toxaemia showed a marked hyperglycaemia (12.4 ± 5.4 mmol/L). Although our findings are based on a small population sample (10 goats), we nonetheless postulate that hyperglycaemia could be explained by the death of the foetuses. Caesarian surgery was performed on four of the five does with hyperglycaemia (HG does). In the fifth, kidding was induced. In this group, two does had two dead foetuses, two had three dead foetuses and one does had four foetuses, only one of which was alive. Caesarian surgery was performed on all five does with hypoglycaemia (LG does). Four does of the LG group had three foetuses and one had two foetuses, all alive. The HG doe had lower rectal temperatures, lower sodium and higher urea nitrogen (BUN) in the blood when compared with the LG does. As the condition of affected does may deteriorate quickly, the results of the present study suggest that in the last days of pregnancy goats with pregnancy toxaemia and concurrent hypoglycaemia should be considered for caesarian surgery.

## Background

The importance of glucose in the pregnant goat (and ewe) as the major source of energy to the foetus(es) is well known. So pregnant goats are at high risk of developing pregnancy toxaemia due to the rapid foetal growth [1]. The energy requirements of the pregnant goat increase by a factor of 1.5 when she carries one foetus and by a factor of 2 when she carries two foetuses [2]. Blood glucose levels in pregnant goats is generally low, because of foetal demand. There is little information in literature addressing the occurrence of hyperglycaemia in pregnant does. Bulgin [3] showed that as the disease progresses in ewes, blood glucose and cortisol levels may be elevated (above 3.85 mmol/L and 10 ng/ml, respectively) due to foetal death. Wastney *et al *[4] suggested that the hyperglycaemia occurs because foetal death removed the suppressing effect of the foetus on hepatic gluconeogenesis. Smith and Sherman [5] referred to the existence of a marked hyperglycaemia in terminal cases.

The main objective of our study is to show that levels of glycaemia in pregnant goats with pregnancy toxaemia could be an indicator of foetuses survivability.

## Results

The data from the physical examination of the 10 does is presented in Table 1 and blood data is shown at Table 2.

**Table 1:** Data from the physical examination of five does with pregnancy toxaemia and hypoglycaemia (LG) and five does with pregnancy toxaemia and hyperglycaemia (HG)

Parameter	LG	HG	Reference values *
Age (years)	3.4 ± 0.9 (2 - 4)	4.6 ± 1.3 (3 - 6)	NA

Rectal temperature C^°^	37.8 ± 1.5 (35.3 - 39.1)	34.3 ± 1.2^y^ (33 - 35.6)	38.8-40

Heart rate (beats/min)	67 ± 36 (36 -128)	85 ± 33 (44 - 116)	70-90

Respiratory rate (breaths/min)	68 ± 39 (36 - 112)	49 ± 21 (28 - 72)	15-30

Rumen activity (contractions/min)	No activity	No activity	1-2

Body condition score	> 4	> 4	2.5 - 3

Outcome	Four dead	All dead	NA

Results are expressed as mean ± standard deviation. The range of values is shown in brackets.

^y ^p < 0.005

* Pugh DG [2]

**Table 2:** Blood data collected from five does with pregnancy toxaemia and hypoglycaemia (LG) and five does with pregnancy toxaemia and hyperglycaemia (HG)

Parameter	LG	HG	Reference values*
Glucose (mmol/L)	1.76 ± 0.5 (1.27 - 2.37)	12.4 ± 5.4^y^ (6.16 - 21)	2.75 - 4.13

pH	7.0 ± 0.2 (6.80 - 7.26)	6.8 ± 0.2 (6.6 - 7.0)	7.32 - 7.5

pCO2 (mmHg)	23.5 ± 5.3 (16 - 30)	30.6 ± 10.1 (18 - 48)	38 - 45

HCO3- (mmol/L)	6.5 ± 3.8 (3.7- 11.3)	5.4 ± 3.8 (2.7 - 11.9)	20 - 25

Na (mmol/L)	140 ± 4 (135 -146)	131 ± 3.4^y^ (128 - 136)	142 - 155

K (mmol/L)	2.8 ± 0.5 (2.0 - 3.2)	3.2 ± 0.5 (2.4 - 3.7)	3.5 - 6.7

Cl (mmol/L)	109 ± 3.3 104 - 113	103 ± 9.3 104 - 113 (n = 4)	99 - 110

BUN (mmol/L)	3.4 ± 2.13 1.5 - 7	8.7 ± 3.35^x^ 4.3 - 12.5 (n = 4)	1.7 - 3.3

Results are expressed as mean ± standard deviation. The range of values is shown in brackets.

^x ^p < 0.05

^y ^p < 0.005

* Pugh DG (2)

When compared with the reference interval, the LG group had two does with low rectal temperature, four does with lower heart rate, one with a high heart rate and all five does with higher respiratory rate. In the HG group, all five does had a lower rectal temperature, one had a lower heart rate, two had a higher heart rate and four had a higher respiratory rate when compared with the reference interval. The average rectal temperature in the HG group was significantly lower than in the LG group (p < 0.05). All the does were recumbent, five of them were able to stand and walk when forced to do so (three LG does and two HG does). The other five were unable to stand or walk (two LG does and three HG does). Three does vocalized when approached (one LG doe and two HG does). Rumen motility was absent in all does. Two does, one in each group, showed swollen limbs (subcutaneous edema). Urine was collected from two does (one LG doe and one HG doe) and analyzed with a dipstick^1^. Both does showed aciduria, ketonuria and the HG doe had additionally glycosuria (the blood glucose of this doe was 21 mmol/L).

The major finding in the blood data was very marked metabolic acidosis (low pH and HCO3^-^) in all 10 does. All except one LG does had Na blood levels below the reference range. All but one HG does had lower pCO2 blood levels. In relation to K only one HG doe had blood levels within the reference range, all the others were below it. Two HG does had lower Cl levels, one had higher levels and one had levels within the reference range. In the LG group one doe had higher levels than the reference range. Finally, all the HG does had higher BUN levels while in the LG group one had higher levels and one had lower levels than the reference range. We were unable to determine the blood Cl and the BUN data in one HG doe due to a failure in the portable analyzer. When the mean levels of the two groups were compared, there was a statistically significant difference between the blood levels of glucose (p < 0.005), Na (p < 0.005) and BUN (p < 0.05) (Table 2).

Following caesarian surgery in the LG group, four were found to have three foetuses and one had two foetuses, all alive, in the HG group, two had two dead foetuses, two had three dead foetuses and one doe had four foetuses, only one of which was alive.

Of the five does with hyperglycaemia, a caesarian surgery was performed on four; the fifth, after kidding was induced by injecting a combination of dexamethasone^5 ^(1 mg/10 Kg BW, IM)) and dexcloprostenol^6 ^(125 μl, IM) delivered two dead fetuses.

## Discussion

The blood sugar level is fairly constant in a given animal when variations, due to circadian rhythm, different breeds, sex, nutrition and stress of handling, are taken into account [6]. But, large differences exist between different animal species. Whereas in humans and other monogastrics, glucose levels of about 5.5 mmol/L are common (rising after a meal to 6.6 to 7.15 mmol/L and falling between meals to 4.4 mmol/L), in ruminants blood glucose levels are usually much lower, approximately 2.2 to 3.3 mmol/L [6,7].

**Figure 1:**
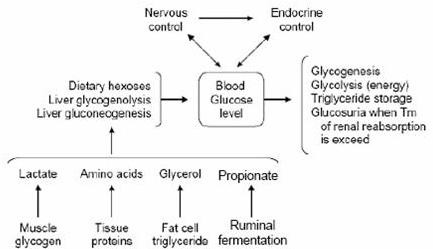
**Regulation of the blood glucose level (adapted from 6)**.

Regulating the blood glucose level is a complex function, under the general control of the neuroendocrine system, in which the liver plays a key role. This regulation can be seen in Figure 1

The last 6 weeks of gestation in goats (and ewes) are a critical period for the pregnant animal because approximately 80% of the foetal growth occurs during this period. Studies in sheep have shown that they synthesize about 100 g a day, but during late pregnancy this basal rate can go up to about 180 g a day [1]. When the rate of synthesis is too low, hypoglycaemia develops and the animal becomes ketotic [1]. Pregnancy toxaemia in small ruminants occurs because of the competition for glucose between the pregnant animal and its foetuses, as these experience very rapid growth [3]. The foetus has developed several strategies in order to minimize the lack of glucose that can occur in the pregnant female. Ovine placenta has the ability to transfer glucose to the foetus even with a very low concentration of maternal blood glucose. The sheep foetus maintains low concentration of plasma glucose (about 0.44 mmol/L), which facilitates transfer of glucose from the maternal plasma. The sheep foetus maintains a relatively high (4.4 - 5.5 mmol/L) plasma fructose concentration as a store for carbohydrate which can be utilized by its tissues. Fructose is synthesized entirely from glucose by the placenta and cannot readily pass through the placenta into maternal circulation. Even though fructose is more abundant than glucose in the plasma of foetal sheep, the foetal tissues utilize about twice as much more glucose than fructose. Foetal tissues have an amazing capacity to remove glucose from plasma at very low concentrations [1].

In all pregnant animals the foetus continuously withdraws metabolites, especially glucose and aminoacids, so it is logical that in small ruminants the main metabolic disorders, or production disease, associated with carbohydrate, fat and protein metabolism is pregnancy toxaemia (usually in multiple pregnancies). Fatty liver is almost always associated with this and is frequently found when necropsies are performed on these animals. It is well known that ruminants are not efficient at transporting lipoproteins out of the liver and back to the adipose tissue [2].

Withdrawal of large quantities of metabolites (as by the foetus in late gestation) can overwhelm the body dam's ability to mobilize metabolic substrates and this leads to pronounced hypoglycaemia. Ketosis of varying intensity occurs associated with hypoglycaemia and metabolic acidosis. Ketosis may result in acidosis because some of the hydrogen ions produced with the ketone bodies remain in the plasma, decreasing blood pH [6].

Obese goats are at greater risk of developing pregnancy toxaemia, especially when they carry twins or triplets. Some authors [5,7,8] refer to this particular pregnancy toxaemia as "fat doe pregnancy toxaemia" (estate ketosis) which is caused by over-conditioning of the flock, herd or individual during early pregnancy. According to Pugh [2] pregnant goats should have a body condition score of between 2.5 and 3 some 45 days before parturition. All ten goats of this study had a body condition scores above 4. This was assessed by palpation of the sternum [9] Goats are considered to be tropical/subtropical animals and body fat stores that develop are laid down not in subcutaneous tissue but in the omentum and the mesentery and around the kidneys. For this reason, conventional body condition scoring based on lumbar palpation can be misleading; another approach is to utilize lumbar scoring in parallel with assessing a score on the same scale by palpating the sternum, and assessing its degree of fat cover [9].

During late gestation, the abdominal space is filled with accumulated fat and an ever-expanding uterus. Because of the lack of rumen space, these females have difficulty consuming enough feedstuff to satisfy their energy requirements [2].

The great accumulation of fat in the abdominal cavity and a very small rumen were consistent findings observed during the necropsies in our study. A marked fatty liver was also observed in three necropsies carried out (two HG doe and one LG doe).

In sheep and goats, pregnancy ketosis is much more common in highly prolific selected breeds [5]. All the does in this study were from the Saanen breed. The mere fact that there were more Saanen goats (1100) on this farm than Alpine goats (600) is probably not a satisfactory explanation for this. We had not (prior to this study) observed more abdominal accumulation of fat in Saanen goats than in Alpines. Therefore we think that the discrepancy is probably better explained by the Saanen breed being more likely to be pregnant with multiple foetuses than are Alpines.

According to Rook [8] clinical cases are typically limited to older goats and ewes during their second or subsequent pregnancies. In our study, the ranges of ages of the does were between 2 and 4 years in the LG group and between 3 and 6 years in the HG group.

Pregnancy toxaemia is a disease characterized by a high mortality rate and on the farm where this study was done is the main cause of does deaths. In our study, only one doe survived (10%).

The diagnosis of pregnancy toxaemia was achieved from the history (last days of pregnancy) and by physical exam and was confirmed by a blood analysis.

Blood levels of glucose in affected animals varied dramatically and this gave rise to the idea that hypoglycaemia might indicate that the foetuses are alive and hyperglycaemia that the foetuses are dead. Because not many practitioners can measure glucose in the blood as we did in our study, this information is not available in the field. It would be much easier if a urine sample could be collected to measure glucose concentration. Glucose is not found in the urine of normal domestic animals unless the blood glucose increases above the renal threshold, which is thought to be around 5.5 to 7.7 mmol/L in ruminants [10]. However, in goats it appears to be more difficult to collect a urine sample than in cows or ewes although there is evidence that they usually urinate when forced to stand up [11].

The withdrawal of large quantities of metabolites can overwhelm the body's ability to mobilize metabolic substrates, and can result in severe hypoglycaemia, variable degrees of ketosis and metabolic acidosis [12]. Ketone bodies (beta-hydroxybutyrate and acetoacetate) are strong acids [1,6,12], and their accumulation in the blood leads to metabolic acidosis (ketoacidosis). The situation can progress to an irreversible stage, where there is dehydration and increased BUN values [12]. According to some authors, this increase in BUN can be caused by increased protein catabolism, by decomposing foetuses or by terminal kidney failure [13]. According to Marteniuk and Herdt [14], pregnancy toxaemia is often accompanied by dehydration, electrolyte abnormalities and renal failure. Experimental models of ketonaemia have shown that systemic hypertension can occur in pregnant ewes after as little as 24 hours of food deprivation. Renal dysfunction begins with the onset of hypertension and results in as much as a 51% decrease in glomerular filtration, as well as being indicated by a rise in BUN values and protein loss in the urine [3]. In our study, the levels of BUN were elevated in the HG does when compared with the LG does (p < 0.05). Kidney failure could explain the lower levels of sodium that also occurred in the HG does (p < 0.005). However, urine samples obtained from two does did not show proteinuria.

A marked hypokalemia could be observed in nine does. In general, changes in the pH of the extracellular fluid produces reciprocal H^+ ^and K^+ ^shifts between the cells and the extracellular fluid. As a result, K^+ ^tends to move into the cells with alkalemia and out of the cells with acidemia. These pH-induced effects, however, are transient and frequently overridden by concurrent variations in other mechanisms that influence K^+ ^transport [15]. Hypokalemia has been described in humans with liver failure, including the acute fatty liver during pregnancy [16]. In human patients with ketoacidosis and ketonuria, there is marked loss of K^+ ^in the urine leading to hypokalemia [15].

Hypokalemia could be explained in part because the goats were not eating and, therefore, their dietary K intake would have been reduced. We suggest that these two mechanisms, acting together, were the cause of the hypokalemia that was observed in this study.

Kidding was induced in one doe in the HG group using dexamethasone and prostaglandin; it is arguable as to whether the hyperglycemia was due to the effect of the dexamethasone. In order to clarify this point, blood was taken from 10 additional doe whose kidding was induced in the previous 48 to 24 hours and blood glucose levels were determined. In these doe, mean glucose levels were 3.2 ± 0.7 mmol/L. This would suggest that corticosteroid did not have an effect in the blood glucose levels in this group. A doe that is carrying 2 or 3 foetuses and with a marked negative energy balance has all its gluconeogenic mechanisms working very efficiently, so the administration of a corticosteroid has a very modest effect, if any, on blood glucose levels. Glucocorticoid blood concentrations are elevated in ovine ketosis [1]. They can inhibit glucose utilization, and force the animal's body to rely more on free fatty acids and ketone metabolism for caloric needs [1]. Prolonged hypoglycaemia can lead to hyperactivity of the adrenal glands, with increased cortisol secretion, insulin activity antagonism and to effective inhibition of maternal glucose use [12].

## Conclusions

The blood levels of glucose in pregnant goats with pregnancy toxaemia can be a good indicator of the viability of the foetuses. As the condition of affected does may deteriorate quickly, the results of the present study, although based on a small sample, suggest that in the last days of pregnancy goats with pregnancy toxaemia and concurrent hypoglycaemia should be considered for caesarian surgery. This will avoid prolonged poor welfare for the doe and its kids. In the future, in order to further strengthen these findings, we would like to confirm the viability of the foetuses by ultrasonography.

## Methods

This study was performed on farm with 1700 dairy goats, 30 miles northeast of Lisbon. The goats were from two breeds: Saanen (1100 animals) and Alpine (600 animals). The goats were housed in confined straw yards, had access to free stalls and were fed a complete ration, *ad libidum*. Their Total Mixed Ration (TMR) consisted of corn silage, rye-grass hay, alfalfa hay, brewer's grain and a concentrate mixture. The feed was distributed once a day. All the adult goats had free access to mineral blocks. One month before kidding they started eating wheat straw *ad libidum *and 1 Kg of concentrate distributed 4 times a day. Nutrient analysis of the TMR for the different groups of goats (high producing milk, low producing goats, dry does and young goats) can be seen in Table 3.

**Table 3:** Nutrient analysis of the TMR for the different groups of goats (high producing milk, low producing goats, dry does and young goats)

Nutrient	High Production Goats	Low Production Goats	Dry Does	Young goats (from 6 months until one month before kidding)
Dry Matter intake (Kg/day)	2.4 - 3	1.8 - 2	1.4-1.5	0.8-0.94

Dry matter %	Max:50	Max:50	80 - 90	40

Forage % DM	40	40	50	

Crude Protein (%)	17-18	16-17	12	12

Crude Fat (%)	5	5	4	3

Crude Fiber (%)	16	17	17	17

Acid Detergent Fiber (%)	19	20	21	21

Neutral Detergent Fiber (%)	28	30	31	32

Starch (%)	15-18	12-14	10 - 15	10 - 20

Ca % DM	0.8 - 0.9	0.75 - 0.8	0.35	0.35

P % DM	0.4 - 0.5	0.35 - 0.4	0.25	0.25

Mg % DM	0.25 - 0.3	0.25 - 0.3	0.2	0.2

Salt % DM	0.4 - 0.5	0.4 - 0.5	0.25	0.25

Milk production in this herd averaged approximately 3 L per goat per day. Machine milking was performed twice daily. In this farm there are three kidding seasons per year, in January, April and October. Each kidding season began on the first day of the month, and continued for 45 days.

Four vaccine products were routinely administered to the goats on this farm: [1] A commercial 7-way clostridial bacterin^2 ^was administered to kids at 60 days of age, and a booster dose given 30 days later. This vaccine is administered to adult goats and bucks twice a year [2]. An autogenous *Corynebacterium pseudotuberculosis *(caseous lymphadenitis) vaccine was administered to kids at 60 days of age, and a booster dose given 30 days later. Subsequently, the vaccine was administered annually to all yearling and adult goats [3]. An autogenous *Mannheimia haemolytica *vaccine was administered to kids at 7 to 15 days of age, and a booster dose administered at 45 days of age. Subsequently, this same vaccine was administered to animals of all ages at 6-month intervals [4]. A commercial *Mycoplasma agalactiae *vaccine^3 ^was administered to all adult goats, one month before the beginning of each kidding season, the young does receiving a booster 3 weeks later.

Twice annually, fecal samples were collected from every pen on the farm, and McMaster's fecal egg counts were performed on those samples. Groups of goats were treated with an anti-helmintic drug, where nematode egg counts of 250 eggs or more per gram of feces were obtained. However, during the conduct of this study, nematode egg counts did not exceed this threshold, and no goats were ever treated for internal parasites. It is common knowledge that dairy goats maintained in dry lot have minimal problems with gastro-intestinal nematodes [2].

This study involved 10 does with clinical signs of pregnancy toxaemia. The does were in two groups, a group of does with hypoglycaemia (LG group) and a group with hyperglycaemia (HG group). A physical exam was performed and a blood sample was taken from the jugular vein and analyzed immediately at the farm with a portable analyser^4 ^for glucose, pH, pCO2, HCO3^-^, Na, K, Cl and BUN.

Of the five HG does, a caesarian surgery was performed on four, the other one delivered two dead foetuses after kidding was induced by injecting a combination of dexamethasone^5 ^(1 mg/10 Kg BW, IM)) and dexcloprostenol^6 ^(125 μl, IM). Caesarian surgery was performed in all five LG does.

This research project was approved by the University Ethics Committee (Comissão de Ética e Bem-Estar Animal da Faculdade de Medicina Veterinária).

## Statistical analysis

A two-tailed T test for independent samples was used to compare the means values of the two groups, LG and HG [17].

## Endnotes

1. Combur^10 ^Test, Roche

2. Miloxan^®^, Merial

3. Agalactivax^®^, Schering-Plough

4. i-Stat, Sensor Devices Incorporated, Waukesha, Wisconsin, USA

5. Vetacort^®^, Vetoquinol

6. Gestavet-Prost^®^, Hipra

## Competing interests

The authors declare that they have no competing interests.

## Authors' contributions

ML did most of the field work (physical examinations, blood collection). RP identified the sick goats and helped with the field work. GS helped to draft and to revise the manuscript. All authors read and approved the final manuscript.
